# Deep learning model to generate patient-specific pulmonary vein isolation lines from successful atrial fibrillation ablation cases: a proof-of-concept study

**DOI:** 10.3389/fcvm.2026.1859430

**Published:** 2026-07-06

**Authors:** Kazuo Sakamoto, Takeshi Tohyama, Hirotake Yokoyama, Tsukasa Watanabe, Tomomi Nagayama, Yasushi Mukai, Shunsuke Kawai, Daisuke Yakabe, Hiroshi Mannoji, Kazuhiro Nagaoka, Atsushi Tanaka, Mitsutaka Yamamoto, Kiyohiro Ogawa, Takeshi Mikami, Shujiro Inoue, Susumu Takase, Kei Inoue, Kazuya Hosokawa, Koji Todaka, Hiroyuki Tsutsui, Kohtaro Abe

**Affiliations:** 1 Department of Cardiovascular Medicine/Coronary Care Unit, Kyushu University Hospital, Fukuoka, Japan; 2 Department of Cardiovascular Medicine, Faculty of Medical Sciences, Kyushu University, Fukuoka, Japan; 3 Centre for Clinical and Translational Research of Kyushu University Hospital, Fukuoka, Japan; 4 Institute for Medical Engineering & Science, Massachusetts Institute of Technology, Cambridge, MA, United States; 5 School of Health Sciences, International University of Health and Welfare, Fukuoka, Japan; 6 Department of Cardiovascular Medicine, Graduate School of Medical Sciences, Kyushu University, Fukuoka, Japan; 7 Department of Cardiology, Japanese Red Cross Fukuoka Hospital, Fukuoka, Japan; 8 Department of Cardiovascular Internal Medicine, Clinical Research Institute, National Hospital Organization Kyushu Medical Center, Fukuoka, Japan; 9 Department of Cardiovascular Medicine, Hamanomachi Hospital, Fukuoka, Japan; 10 Department of Cardiovascular Medicine, St. Mary’s Hospital, Fukuoka, Japan; 11 Division of Cardiology, Cardiovascular and Aortic Center, Saiseikai Fukuoka General Hospital, Fukuoka, Japan; 12 Department of Cardiovascular Medicine, Harasanshin Hospital, Fukuoka, Japan; 13 Department of Cardiology, Fukuoka City Hospital, Fukuoka, Japan; 14 Department of Cardiology, Munakata Suikokai General Hospital, Fukuoka, Japan; 15 Department of Cardiology, Aso Iizuka Hospital, Fukuoka, Japan; 16 SUSMED, Inc., Tokyo, Japan

**Keywords:** 3D voltage map, artificial intelligence, atrial fibrillation, catheter ablation, pulmonary vein isolation

## Abstract

Pulmonary vein isolation (PVI) is an established standard ablation for atrial fibrillation (AF), however, AF recurrence remains a major clinical challenge. We developed a deep learning model to generate patient-specific PVI lines on pre-ablation 3D voltage maps, using lesion sets from successful AF ablation cases with documented freedom from recurrence for more than one year as ground truth. Using a U-Net-based architecture trained and evaluated on 513 maps from 171 such cases, the model reproduced the anatomical and electrophysiological features of these PVI lesion sets. On the held-out test set, the model achieved a mean Intersection over Union of 0.87 and a Dice score of 0.93. As a proof of concept, these findings suggest that the model can reproduce patient-specific PVI patterns associated with successful outcomes; whether this translates into reduced recurrence requires prospective clinical validation.

## 1 Introduction

Pulmonary vein isolation (PVI) is an established standard ablation for atrial fibrillation (AF), however, AF recurrence following ablation remains a major clinical challenge (1). The primary causes of recurrence are PV reconnection and untreated non-PV triggers. Although some non-PV triggers originate from the PV antrum and a wider PVI area may reduce AF recurrence, the optimal isolation area depends on the operator's skill and the anatomy of the left atrium (LA) in each patient (2). Recently, the advent of pulsed field ablation (PFA) has minimized the risk of collateral damage, enabling safer PVI with more flexible and extensive lesions compared to those produced by conventional thermal ablation modalities, such as radiofrequency and balloon ablation (3, 4). Thus, PFA allows the creation of an adequate PVI area without increasing complications known to be associated with thermal ablation. Concurrently, deep learning has demonstrated remarkable progress in cardiovascular medicine, in both signal processing and imaging data analysis (5, 6). AI-driven approaches are being actively explored across cardiac electrophysiology. A recent comprehensive review summarized applications spanning arrhythmia detection, electroanatomical mapping, ablation target identification, and outcome prediction (7, 8). The present study addresses a complementary aspect by generating patient-specific PVI lines from the pre-ablation voltage map.

## 2 Methods

### 2.1 Study design and patient cohort

In this retrospective, multicenter study, data were collected from 1,969 consecutive patients who underwent catheter ablation for AF between April 2016 and March 2023 at Kyushu University Hospital and eight collaborating institutions (8). High-density 3D voltage maps of the left atrium (CARTO 3 System, Biosense Webster, Inc.) providing both anatomical and electrophysiological information, were acquired for all patients. This study was approved by the Institutional Review Board of Kyushu University and conducted in accordance with the Declaration of Helsinki. This study was reported in accordance with the Transparent Reporting of a multivariable prediction model for Individual Prognosis Or Diagnosis + Artificial Intelligence (TRIPOD + AI) statement, and a completed TRIPOD + AI checklist is provided as Supplementary Material. As this is a model-development proof-of-concept study without external validation, the development-relevant items of the checklist apply.

### 2.2 AI model architecture, training, and performance metrics

**Figure 1:**
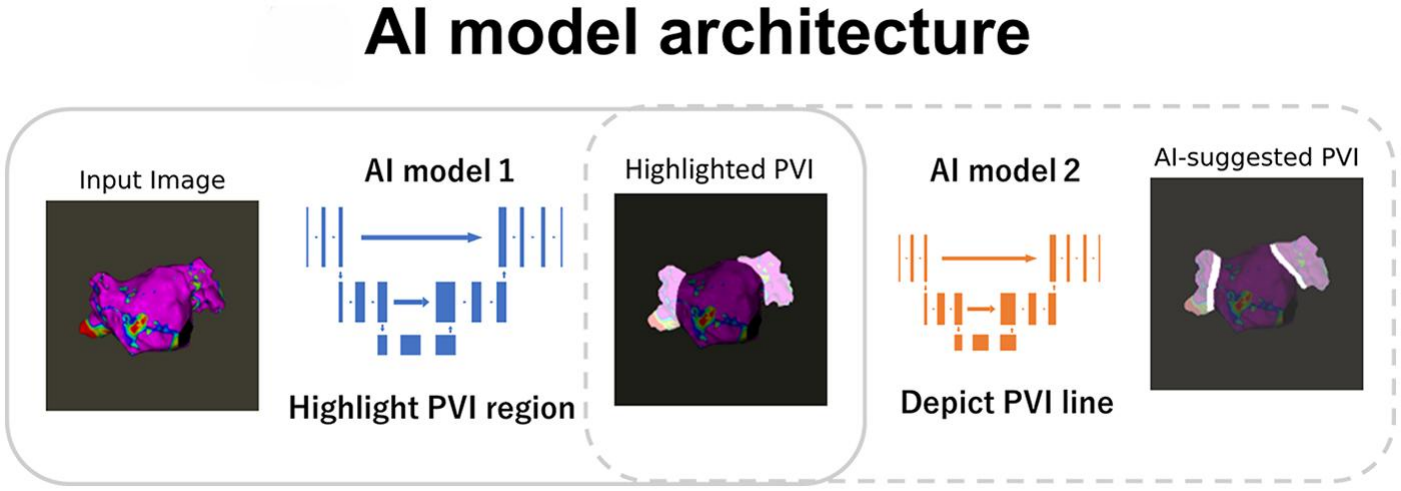
AI model architecture. The deep learning model is based on the U-Net architecture. The workflow consists of two stages: AI Model 1 identifies and highlights the target PVI region from the pre-ablation 3D voltage map, and AI Model 2 generates the specific PVI lines within that region.

The input to the model consisted of 2D screen captures of the pre-ablation 3D voltage map at three standardized anatomical projections: anterior-posterior (AP), posterior-anterior (PA), and superior (SUP) views, exported from the CARTO 3 System under fixed color-scale and window settings to ensure a consistent voltage-to-color mapping across patients. Each patient contributed three input images (one per projection). Ground-truth annotations were created by overlaying actual treatment records on the 3D voltage map, and both the PVI region and PVI line were drawn as region masks. This was performed by a single electrophysiologist with confirmation by multiple operators as needed; formal quantitative assessment of inter-operator variability was not performed. As shown in Figure 1, the model architecture is centered on a U-Net (9) (AI Model 1) that segments the candidate PVI region from the pre-ablation voltage map; this region prediction is the principal output of the study. For visualization of the lesion boundary, a second U-Net of identical architecture (AI Model 2) generates the PVI lines themselves, taking as input the voltage map overlaid with the PVI region mask (ground-truth during training and evaluation). Both modules adopted a seven-level encoder–decoder architecture and were trained and evaluated independently using the same procedure. The input is 512 × 512 × 3 (RGB image) and the output is 512 × 512 × 2 (SoftMax, 2-class classification). The loss function was a weighted combination of categorical cross-entropy and soft IoU loss, both computed only within the atrium region to exclude background pixels. Models were trained in two phases using the Adam optimizer: (1) pre-training on all available training images for 30 epochs with a learning rate of 1 × 10⁻³, followed by (2) fine-tuning on recurrence-free cases for up to 40 epochs with a learning rate of 1 × 10⁻⁴. The batch size was 4, and early stopping with a patience of 15 epochs was applied during fine-tuning. Data augmentation comprising horizontal and vertical flipping and 90°/180°/270° rotation was applied during training. A 5-model ensemble with the same set of transformations applied as test-time augmentation was used for final predictions. The dataset was split at the patient level into a training set and a held-out test set in an 80:20 ratio; all projections from a given patient were assigned exclusively to one set to prevent patient-level leakage. As this is a proof-of-concept study, a separate validation set was not used and hyperparameters were set based on preliminary experiments. Performance was evaluated with the intersection over union (IoU) and Dice coefficient, calculated within the atrium region as the pixel-wise overlap between the AI-predicted and ground-truth binary segmentation masks for the positive class (isolated region for AI Model 1; PVI line for AI Model 2):

$$\mathrm{IoU}=\frac{TP}{TP+FP+FN}$$

$$\mathrm{Dice}=\frac{2\times TP}{2\times TP+FP+FN}$$

where TP, FP, and FN denote true positive, false positive, and false negative pixels, respectively. All experiments used TensorFlow 2.17.0 on an NVIDIA RTX A6000. Source code is available at https://github.com/ttohya/AI-PVI.

## 3 Results

**Figure 2:**
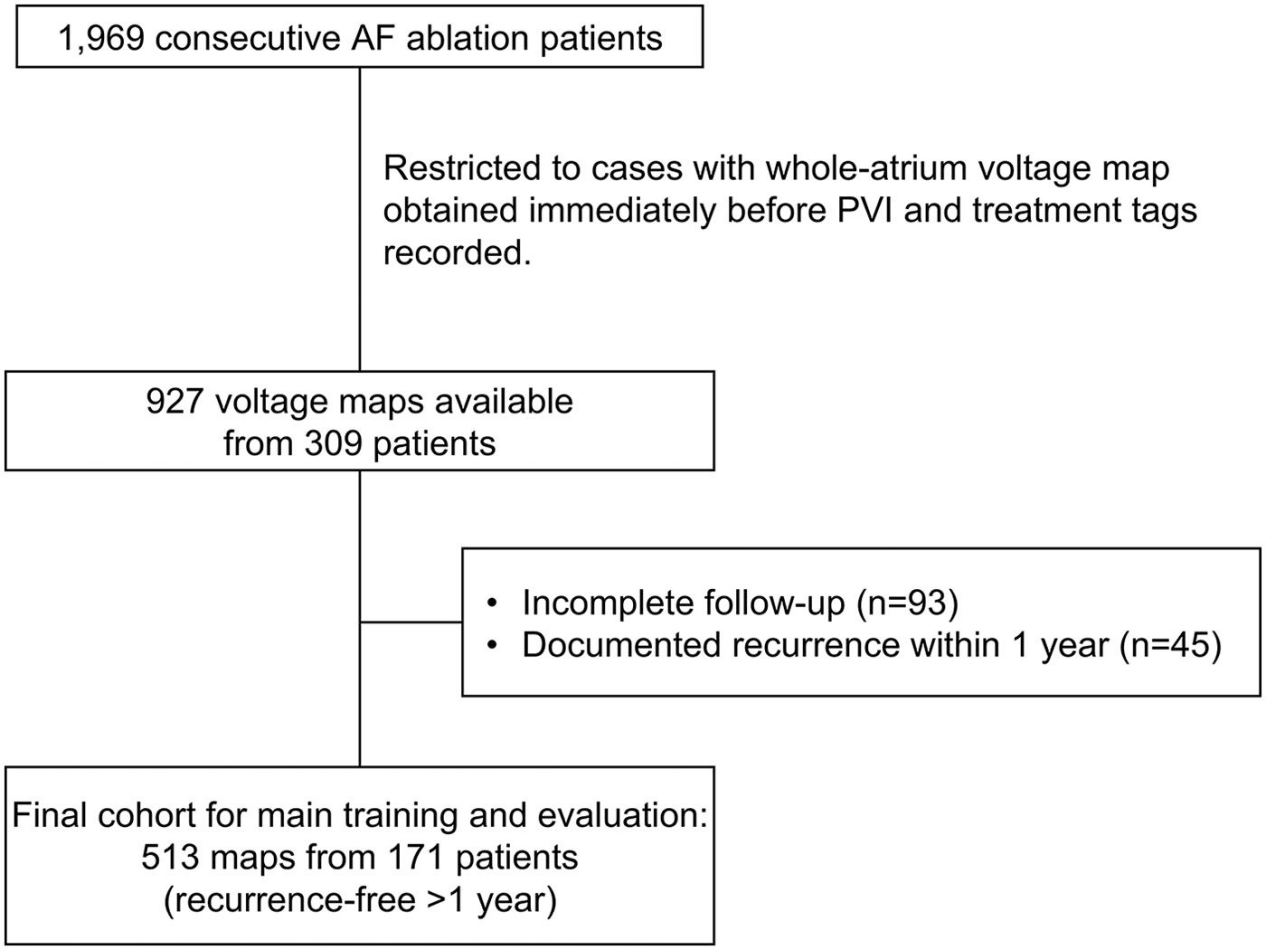
Data flow diagram of AI models. Pre-training used all maps except the held-out test set (the two excluded patient groups plus the training split of the final cohort). The final cohort of 513 maps from 171 patients was split 80:20 for training and model evaluation, and the model was fine-tuned and evaluated on these splits.

Of 1,969 consecutive AF ablation patients, 309 had an eligible pre-PVI whole-atrium voltage map (927 maps). Of these, 138 patients (414 maps) were excluded from the final analytic cohort: 93 with incomplete follow-up and 45 with documented recurrence within 1 year. The final analytic cohort comprised 171 patients (513 maps) with documented freedom from atrial arrhythmia for more than 1 year (Figure 2). All maps except the held-out test set, that is, the 414 maps from excluded patients together with the training split of the final cohort, were used for pre-training, and the model was then fine-tuned and evaluated on the 80:20 split of the final cohort. The baseline characteristics of the 171-patient cohort used for AI modeling were representative of the general AF ablation population (Table 1). The median age was 72 years, and 52.6% of patients had paroxysmal AF. On the test set, AI Model 1, which segments the candidate PVI region, achieved an IoU of 0.87 (95% CI: 0.86–0.88) and Dice of 0.93 (95% CI: 0.92–0.94). AI Model 2, which generates the PVI lines themselves, achieved an IoU of 0.80 (95% CI: 0.79–0.81) and Dice of 0.89 (95% CI: 0.88–0.90).

**Table 1:** Patient characteristics.

Features	171 patients (513 Maps)
Age, years (IQR)	72 (64–78)
Female, *n* (%)	66 (38.6)
BMI, kg/m^2^ (IQR)	23.2 (21.3–25.7)
PAF, *n* (%)	90 (52.6)
Organic heart disease, *n* (%)	44 (25.7)
Heart failure, *n* (%)	43 (25.1)
Hypertension, *n* (%)	96 (56.1)
Diabetes mellitus, *n* (%)	26 (15.2)
History of stroke or TIA, *n* (%)	28 (16.4)
Left atrial diameter, mm (IQR)	42 (36–45)
Left ventricular ejection fraction, % (IQR)	64.3 (58.4–69.8)
Antiarrhythmic drugs at discharge, *n* (%)	100 (58.5)

Values are presented as *N* (%), median [Interquartile range (IQR)].

## 4 Discussion

### 4.1 Model innovation and impact on prediction

This study developed a deep learning model to reproduce PVI lines from AF ablation cases with documented freedom from recurrence for more than one year. While anatomical recommendations for optimal PVI extent have been discussed (2, 10), there is little prior research on prediction models that recommend specific line drawing. The primary innovation of our model lies in its training methodology, which utilizes a “ground truth” dataset derived exclusively from successful cases—patients who remained free from atrial arrhythmia for more than one-year post-ablation. Traditionally, determining PVI lines has been a subjective process that is heavily dependent on the operator's experience and the patient's specific anatomy. By leveraging the U-Net architecture, our model identified complex correlations between preprocedural voltage patterns and isolated area on the post-ablation voltage map from successful cases. This enables objective, data-driven prediction of patient-specific PVI lines that have been dependent on clinician experience and difficult to define explicitly.

### 4.2 High accuracy and qualitative validity

Validation on the test set yielded an IoU of 0.87 (95% CI: 0.86–0.88) for AI Model 1, indicating that the predicted regions closely matched the ground-truth lesion sets derived from cases with documented freedom from recurrence for more than one year. Because these reference lesion sets represent procedures associated with durable freedom from atrial arrhythmia, this suggests that the model can generate patient-specific PVI plans that reproduce lesion patterns characteristic of successful ablation. Whether reproduction of these patterns translates into reduced recurrence in clinical practice remains to be evaluated in prospective studies.

### 4.3 Clinical implications

With regard to the clinical relevance of these findings, the intended role of the model is decision support at the procedure-planning stage. By presenting a patient-specific candidate isolation area, the model could serve as a quantitative reference that an operator compares against their intended wide antral circumferential ablation (WACA) line. In patients whose left atrial anatomy or substrate makes the WACA line uncertain, such a reference might prompt reconsideration of the planned line and could help standardize the isolation area across operators of differing experience.

A recent randomized controlled trial demonstrated that AI-based analysis of spatio-temporal dispersion patterns in electrograms can identify additional ablation targets beyond PVI and improve outcomes in persistent AF (11). Although our study addresses a different aspect—the generation of PVI lines themselves—this supports the broader feasibility of AI-assisted ablation planning. Integration into 3D mapping systems could shorten the learning curve for less experienced operators and streamline the procedural workflow. Although the present model was developed using radiofrequency ablation cases and was not evaluated for PFA, the flexible lesion sets it predicts might in principle be relevant to other energy sources, including PFA; this remains a question for future work.

### 4.4 Limitations

There were several limitations as follows. First, the validation presented here is retrospective and technical, assessing the agreement of the predicted lesion sets with historical ground-truth lesion sets rather than their effect on patient outcomes; the model has not been clinically validated and is therefore not ready for clinical use. Its clinical efficacy and any superiority over conventional planning remain to be established in prospective studies, and the present results should be regarded as exploratory evidence of technical feasibility rather than evidence of clinical effectiveness. Second, our study did not include PFA cases, which is a standard ablation modality; therefore, validation in PFA cohorts is necessary. Third, the ground truth was defined as the lesion set delivered to patients who remained free from atrial arrhythmia for more than one year. Because the lesion sets used as ground truth come only from successful cases, they are a valid but incomplete reference: they show patterns that worked, not that a given pattern was responsible for the outcome. Furthermore, freedom from recurrence is determined by patient, substrate, pharmacological, and procedural factors collectively, so the ground-truth patterns reflect only a subset of lesion configurations that may lead to successful outcomes. The model therefore learns lesion patterns associated with successful outcomes in our cohort and should not be interpreted as identifying the unique optimal design. Fourth, in keeping with its retrospective, proof-of-concept design, the study did not include repeat electroanatomical mapping during follow-up; freedom from recurrence was therefore based on clinical follow-up rather than confirmed electrically, and the durability of isolation or the site of any pulmonary vein reconnection was not directly examined. Likewise, the analysis was not designed as a comparative study and did not include recurrence cases or a head-to-head comparison of AI-guided vs. conventional planning, so the relative efficacy of the predicted lesion sets remains to be established in prospective work. Finally, although the dataset was split at the patient level to prevent patient-level leakage, it was not partitioned by center, operator, or procedural era. Cases sharing the same center, operator, or time period may therefore be present in both the training and test sets, and the reported performance may not fully reflect generalisability to unseen centers or operators. External validation in independent cohorts is warranted.

## 5 Conclusion

We developed a deep learning model that accurately generates patient-specific PVI lines derived from AF ablation cases with documented freedom from recurrence. This proof of concept marks an encouraging step toward data-driven precision ablation planning, providing a strong foundation for the prospective studies that will establish its clinical benefit. However, the model has not yet been clinically validated, and prospective clinical evaluation is required before it can be used to inform patient care.

## Data Availability

The raw data supporting the conclusions of this article will be made available by the authors, without undue reservation.
