# Supplementary material for: Deep learning model to generate patient-specific pulmonary vein isolation lines from successful atrial fibrillation ablation cases: a proof-of-concept study
Source: Front Cardiovasc Med. 2026 Jul 6;13:1859430. doi: 10.3389/fcvm.2026.1859430 (PMC13381618; doi:10.3389/fcvm.2026.1859430)
Supplement: Supplementary file 1 [file Table1.docx]

**TRIPOD+AI Checklist (completed)**

*Deep Learning Model to Generate Patient-Specific Pulmonary Vein Isolation Lines from Successful Atrial Fibrillation Ablation Cases: A Proof-of-Concept Study*

| **Item** | **Checklist item (abbreviated)** | **Reported in (section)** | **Notes** |
| --- | --- | --- | --- |
| **Title and abstract** | | | |
| 1 | Title: identify study as developing/evaluating a multivariable prediction model, the target population, and the outcome. | Title page | *Title states a proof-of-concept development study.* |
| 2 | Abstract: structured summary (per TRIPOD+AI for Abstracts). | Abstract | *Reports data source/size, U-Net method, IoU/Dice with the proof-of-concept caveat.* |
| **Introduction** | | | |
| 3 | Background: healthcare context and rationale, with references to existing models. | Introduction | *n/a* |
| 4 | Objectives: study objectives; development and/or validation. | Introduction (final sentence) | *Development (proof of concept) stated.* |
| **Methods** | | | |
| 5a | Source of data and rationale; representativeness. | Methods 2.1 | *Retrospective multicentre CARTO-3 voltage-map dataset.* |
| 5b | Dates of participant data (accrual start/end; follow-up). | Methods 2.1 | *April 2016 – March 2023.* |
| 6a | Study setting; number and location of centres. | Methods 2.1 | *Kyushu University Hospital + 8 collaborating institutions.* |
| 6b | Eligibility criteria for participants. | Methods 2.1; Results; Figure 2 | *Eligible pre-PVI whole-atrium map; recurrence-free >1 year.* |
| 6c | Treatments received and how handled. | Methods 2.1; Table 1 | *Catheter ablation (RF); antiarrhythmic drugs at discharge reported.* |
| 7 | Data preparation / pre-processing and quality checking. | Methods 2.2 | *Standardised AP/PA/SUP projections; fixed colour-scale/window; 512×512 RGB.* |
| 8a | Choice of initial predictors / pre-selection. | Methods 2.2 | *Image inputs: pre-ablation voltage maps at three projections.* |
| 8b | Define all predictors; how/when measured. | Methods 2.2 | *n/a* |
| 8c | Blinding of predictor assessment. | n/a | *Not applicable: predictors are exported map images.* |
| 9a | Define outcome predicted; how/when assessed; rationale. | Methods 2.2; Results | *Target = ground-truth PVI region/line mask from recurrence-free cases.* |
| 9b | Blinding of outcome assessment. | Methods 2.2 | *Ground-truth annotated by one electrophysiologist with multi-operator confirmation; inter-operator variability not formally quantified.* |
| 10 | Sample size: how arrived at; justification of sufficiency. | Methods 2.2; Figure 2 | *Not formally powered (proof of concept);* |
| 11 | Missing data: how handled. | Methods/Results; Figure 2 | *Patients with incomplete follow-up excluded from the analytic cohort (flow diagram).* |
| 12a | How data used for development/evaluation (train/tune/test). | Methods 2.2 | *Patient-level 80:20 split; pre-training + fine-tuning.* |
| 12b | How predictors handled in analyses. | Methods 2.2 | *n/a* |
| 12c | Model type, model-building, hyperparameter tuning, internal validation. | Methods 2.2 | *U-Net (7-level encoder–decoder), Adam, LR/epochs/batch, early stopping, augmentation, 5-model ensemble + TTA. No separate validation set (stated).* |
| 12d | Heterogeneity across clusters (see TRIPOD-Cluster). | n/a | *Not applicable: clustered analysis not performed.* |
| 12e | Measures/plots used to evaluate performance. | Methods 2.2 | *IoU and Dice (pixel-wise, within atrium).* |
| 12f | Model updating (e.g., recalibration). | n/a | *Not applicable (development study).* |
| 13 | Class imbalance: methods, if used. | Methods 2.2 | *Loss computed only within the atrium region (background excluded); weighted cross-entropy + soft-IoU. No resampling/SMOTE used.* |
| 14 | Fairness: approaches used and rationale. | n/a | *Formal subgroup/fairness analysis not performed; cohort characteristics reported.* |
| 15 | Model output: output type; thresholds and rationale. | Methods 2.2 | *512×512×2 SoftMax pixel-wise segmentation (2-class).* |
| 16 | Differences between development and evaluation data. | n/a | *Not applicable: no external evaluation cohort.* |
| 17 | Ethical approval and consent/waiver. | Methods 2.1; Ethics statement | *IRB, Kyushu University (approval 21112); Declaration of Helsinki.* |
| **Open science** | | | |
| 18a | Funding source and role of funders. | Funding | *JSPS KAKENHI JP25K11413.* |
| 18b | Conflicts of interest / financial disclosures. | Conflict of Interest | *None declared.* |
| 18c | Study protocol: where accessible / not prepared. | n/a | *No separate prospective protocol; retrospective study.* |
| 18d | Registration: registry/number or not registered. | n/a | *Not registered (retrospective development study).* |
| 18e | Data sharing. | Data Availability | *Available from corresponding author on reasonable request (ethical restrictions).* |
| 18f | Code sharing. | Methods 2.2 | *Code available: https://github.com/ttohya/AI-PVI* |
| **Patient and public involvement** | | | |
| 19 | Patient/public involvement in design, conduct, reporting, interpretation, dissemination: or state none. | n/a | *No patient or public involvement in this retrospective study.* |
| **Results** | | | |
| 20a | Flow of participants (with/without outcome; follow-up). | Results; Figure 2 | *Flow diagram: 1,969 → 309 (927 maps) → 171 patients (513 maps).* |
| 20b | Characteristics of participants (demographics, predictors, missing data). | Results; Table 1 | *n/a* |
| 20c | For evaluation: comparison with development data. | n/a | *Not applicable: no separate evaluation cohort.* |
| 21a | Number of participants and outcome events per analysis. | Results; Figure 2 | *171 patients / 513 maps; patient-level 80:20 split.* |
| 21b | Unadjusted association of each candidate predictor with outcome. | n/a | *Not applicable: image-based segmentation (no tabular predictors).* |
| 22 | Present the final model to allow predictions for individuals. | Methods 2.2; code repository | *Architecture described; trained model/evaluation code released on GitHub.* |
| 23 | Model performance with confidence intervals, incl. key subgroups. | Results | *IoU/Dice with 95% CIs for both models. Subgroup performance not reported.* |
| 24 | Results of any model updating. | n/a | *Not applicable (development study).* |
| **Discussion** | | | |
| 25 | Overall interpretation, including fairness, vs objectives and prior studies. | Discussion 4.1–4.3 | *n/a* |
| 26 | Limitations (representativeness, sample size, overfitting, missing data) and effects on bias, uncertainty, generalizability. | Discussion 4.4 | *Expanded: ground-truth limitation, no remapping, no comparator, lack of clinical validation, leakage by centre/operator/era.* |
| 27a | How poor/unavailable input data are assessed/handled at implementation. | Discussion 4.3–4.4 | *Input is a standardised CARTO export used as a planning aid; addressed partially.* |
| 27b | User interaction and level of expertise required. | Discussion 4.3 | *Decision-support role; the operator compares the predicted area against their intended line* |
| 27c | Next steps for future research; applicability/generalizability. | Discussion 4.3–4.4; Conclusion | *Prospective clinical validation, PFA cohorts, external validation.* |
